# Cortical morphology and cognitive impairments in adolescents with complex congenital heart disease

**DOI:** 10.1093/braincomms/fcag124

**Published:** 2026-04-09

**Authors:** Asuka Toyofuku, Melanie Ehrler, Oliver Kretschmar, Beatrice Latal, Ruth O’Gorman Tuura

**Affiliations:** Child Development Center, University Children’s Hospital Zurich, 8008 Zurich, Switzerland; Children’s Research Center, University Children’s Hospital Zurich, 8008 Zurich, Switzerland; Center for MR Research, University Children’s Hospital Zurich, 8008 Zurich, Switzerland; Child Development Center, University Children’s Hospital Zurich, 8008 Zurich, Switzerland; Children’s Research Center, University Children’s Hospital Zurich, 8008 Zurich, Switzerland; Department of Forensic and Neurodevelopmental Sciences, Institute of Psychiatry, Psychology and Neuroscience, King’s College London, London SE5 8AB, UK; Department of Surgery, Pediatric Cardiology, Pediatric Heart Center, University Children’s Hospital Zurich, 8008 Zurich, Switzerland; Child Development Center, University Children’s Hospital Zurich, 8008 Zurich, Switzerland; Children’s Research Center, University Children’s Hospital Zurich, 8008 Zurich, Switzerland; University Research Priority Program (URPP), Adaptive Brain Circuits in Development and Learning (AdaBD), University of Zurich, 8057 Zurich, Switzerland; Center for MR Research, University Children’s Hospital Zurich, 8008 Zurich, Switzerland

**Keywords:** brain volume, surface area, cortical thickness, gyrification, MRI

## Abstract

Individuals with complex congenital heart disease (CHD) are at increased risk for cognitive impairments linked to altered brain structure, including reduced cortical volume. Cortical volume can be interpreted in relation to other morphological properties, such as cortical thickness (CT), surface area (SA) and cortical folding (gyrification index: GI). CT and SA exhibit distinct developmental, genetic and evolutionary origins, while cortical folding emerges from developmental and mechanical processes associated with cortical SA expansion. Exploring these individual cortical morphometric changes may provide additional insight into cortical volume changes in CHD and their relationships with cognitive function. High-resolution 3D T1-weighted images, intelligence quotient (IQ) and executive function (EF) scores were acquired from a final sample of 49 patients with CHD (mean age = 13.65, male: 63%) and 80 controls (mean age = 12.86, male: 49%). Cortical reconstruction and volumetric analyses were performed using Freesurfer version 7.1. Group differences in cortical volume, total SA, mean CT and mean GI, as well as their associations with cognitive measures, were investigated through vertex-wise multivariate general linear modelling. Age, sex and parental education level were adjusted in all analyses. While both SA and CT were significantly reduced in the CHD group, both globally (*P* < 0.001) and regionally, the CHD group exhibited higher and lower local GI than controls in several regions, while showing no statistically significant difference in mean GI (*P* = 0.27). Among the three cortical measurements, SA was the most widely affected across the brain, and total SA showed a stronger and more consistent correlation with cortical volume (*R*^2^ = 0.91) than mean CT (*R*^2^ = 0.30) or mean GI (*R*^2^ = 0.38). Furthermore, SA and GI, but not CT, were widely associated with IQ and EF across the brain. These relationships were especially prominent in the frontal and occipitotemporal cortices, where patients with CHD exhibited stronger associations between SA and cognitive performance compared to controls. These findings indicate that cortical volume reductions observed in CHD primarily reflect reduced cortical SA rather than cortical thinning or altered cortical folding. While structure–function associations between cortical morphology and cognition are established in healthy populations, our data suggest that these relationships are accentuated in CHD, likely due to disrupted neurodevelopmental processes. This study underscores the importance of differentiating cortical morphometric features to improve our understanding of brain–behaviour associations and the neurobiological underpinnings of cognitive impairment in CHD.

## Teaser

Toyofuku *et al*. investigated the cortical morphology in adolescents with congenital heart disease (CHD). They found that surface area and gyrification were widely affected by CHD and were strongly associated with cognitive function, whereas the impact of CHD on cortical thickness (CT) and the functional relationship of CT was minimal.

## Graphical Abstract

**Graphical Abstract:**
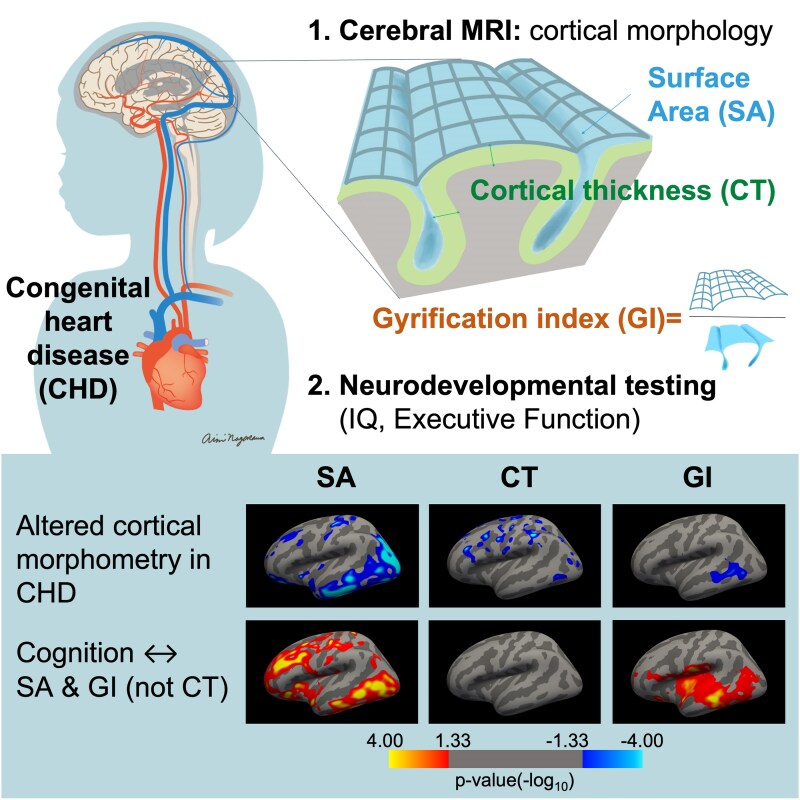

## Introduction

Congenital heart disease (CHD) refers to a range of structural abnormalities of the heart or its major blood vessels, affecting ∼8 per 1000 live births.^1^ Due to altered brain perfusion, chronic hypoxia, brain injuries and many other clinical risk factors, individuals with CHD are often reported to have a spectrum of cognitive impairments together with altered brain structures—ranging from total and regional grey/white matter volume reduction, white matter microstructure integrity and structural connectivity.^2,3^ Structural imaging studies have suggested that populations with CHD already show smaller brain volumes during the foetal period,^4-9^ throughout the neonatal and postnatal periods,^10-17^ and this gap persists until adulthood.^18-21^ A reduction in cortical volumes, along with reductions in other brain regions, was reported in the majority of these studies.

However, the cortical volume, which the majority of CHD brain-imaging studies assessed, can be interpreted in relation to other morphological properties that describe cortical organization, such as cortical thickness (CT), surface area (SA) and cortical folding. CT is defined as the distance between the pial surface and the white matter surface, while SA is the area of the pial surface covering all gyri and sulci. CT and SA are suggested to be evolutionarily distinct^22,23^ and controlled by different genetic variants,^24,25^ highlighting their independent developmental trajectories. Cortical folding, on the other hand, enables more cortical SA to be compacted into a limited cranial volume. It is thought to emerge from a combination of spatio-temporal neuronal growth patterns, as well as the rapid expansion of the SA during development.^26-28^ Cortical folding is typically indexed by the gyrification index (GI), which represents the extent to which the cortex is folded inward relative to the exposed cortical SA.^29^

Given that volumetric findings could reflect any combination of altered CT, SA^30^ or GI, investigating these cortical morphometrics together can provide additional insight into individual differences in brain structure^31^ and may shed light on the mechanistic underpinnings of brain alterations in patients with CHD.

So far, morphometric studies on patients with CHD are relatively scarce, but a few studies have been conducted in foetuses,^5,32^ neonates^16,33-37^ and adolescents.^38,39^ They mostly reported a reduction of total SA or alterations in GI, although one foetal study reported no CHD-control difference in total SA and GI,^32^ and other studies of neonates^35^ and adolescents^38^ with CHD noted that cortical folding was not affected.^35^ In adolescents, one study investigated SA in a lobe-based manner, reporting statistically significant SA reductions for the CHD group in temporal and parietal lobes, less so in frontal lobes, but not in occipital lobes.^20^ In contrast to the relatively consistent reports on reduced total SA, findings on mean CT in CHD are conflicting. One study in neonates^16^ and another in adolescents^20^ found no statistically significant group difference in mean CT, whereas other adolescent/adult studies found a significantly decreased mean CT^38^ or a widespread bilateral decrease in regional CT.^40^

To date, whole brain analyses of regional SA, CT and GI changes in adolescents with CHD are scarce, and little is known about how volume reductions are associated with altered SA, CT, or GI in these populations. Furthermore, the cortical structure–function relationship and the distinct roles played by CT, SA and GI in cognitive function in CHD are under-researched. Among the two CHD studies performed to date, one reported that low intelligence quotient (IQ) was associated with reduced CT in the left precuneus and the right caudal middle frontal cortex.^41^ The other identified reduced SA in the lateral sulcus is connected with worse executive function (EF) and increased CT and GI in a wide range of regions linked with worse outcomes.^38^

Therefore, this paper aims to scrutinize regional cortical alterations in adolescents with CHD, and to assess how SA, CT and GI alterations are associated with cortical volume alterations. Furthermore, we will investigate how individual cortical metrices are linked to cognitive functions. We hypothesize a widespread significant reduction in both SA, CT and GI in individuals with CHD, but we can infer that alterations in cortical volume are more linked with SA rather than CT, since in healthy populations, individual differences in cortical volume are primarily attributed to variability in SA, not CT.^42^ We also expect to see strong associations between SA/CT/GI and cognitive functions, as previous studies on structure–function relationships in healthy populations have consistently shown positive associations between SA and cognition, GI and cognition,^43-45^ as well as age-dependent associations between CT and cognition.^46-48^

## Materials and methods

### Participants

This analysis is part of a broader cohort study (TeenHeart Study^49^). Data were gathered at the University Children’s Hospital of Zurich from April 2019 to September 2021. The recruitment procedure is outlined in the aforementioned study protocol.

Adolescents with complex CHD were eligible for this study if they had undergone cardiopulmonary bypass (CPB) surgery between 2004 and 2012 at the University Children’s Hospital Zurich. Additional inclusion criteria were: (i) undergoing CPB surgery in the first year, (ii) no diagnosis of a genetic or dysmorphic syndrome and (iii) being aged between 10 and 15 years at the time of evaluation. A total of 100 adolescents with CHD participated in the study (56% participation rate) from 178 eligible candidates. Additionally, 104 healthy adolescents aged 10–15 years were recruited as a control group. Exclusion criteria for the control group included: (i) birth before 37 weeks of gestation and (ii) diagnosis of a neurological or significant developmental disorder (e.g. learning disability or attention deficit hyperactivity disorder).

Parental education was measured using a six-point Likert scale (1 = no high school degree, 2 = high school degree, 3 = apprenticeship, 4 = higher diploma for craftsmen/craftswomen, 5 = advanced diploma of higher education, 6 = university degree), and the highest education level of both parents was summed up, with possible parental education scores ranging from 2 to 12.^50^ Clinical risk factors for patients were acquired from their clinical records, such as the length of intensive care unit (ICU) stay, surgery-related parameters and history of stroke/seizure.

Ethical approval was obtained from the ethics committee of the Canton of Zurich. Written informed consent was secured from the legal guardians of all participants and the participants themselves if they were 14 years of age or older.

### Neuropsychological assessment

#### Intelligence quotient

IQ was assessed with a corrected short version of the Wechsler Intelligence Scale for Children, 4th edition (WISC-IV). This short version included the subtests *Matrices, Similarities, Letter Number Sequencing* and *Symbol Search*.^51,52^

#### Executive functions

A comprehensive neuropsychological test battery was used to assess various domains of EFs across working memory, inhibition, cognitive flexibility, planning and fluency.^49^ The Delis–Kaplan Executive Function System,^53^ the Test of Attentional Performance (TAP)^54^ and the Regensburger Verbal Fluency Test^55^ were employed. Detailed descriptions of neuropsychological test measures of EF can be found in Supplementary Table 1. A summary score for overall EF performance was derived using the control group as the normative reference, as previously described.^49,56^ To minimize the effects of fatigue and reduced motivation, tests were administered in a randomized order. The assessments were conducted and interpreted by trained psychologists and paediatricians.

### MRI acquisition

Cerebral MRI data were collected using a 3T GE MR750 scanner. Suitability for MRI acquisition was assessed prior to study participation using a safety screening questionnaire, which was filled out by the parents. All surgery reports were screened for ferromagnetic implants to confirm MR safety. For the MRI scan, hearing protection (earplugs and headsets) was provided.

High-resolution three-dimensional T1-weighted images were acquired using a three-dimensional spoiled gradient echo pulse sequence (SPGR) and were reviewed by radiologists for the presence of macroscopic lesions or other abnormalities. SPGR images were acquired using the following parameters: repetition time/echo time (TR/TE) = 11/5 ms; inversion time = 600 ms; flip angle = 8°; reconstructed matrix = 256 × 256; field of view (FOV) = 26 cm; 176 contiguous axial slices, 1 mm slice thickness.

### MRI data pre-processing

To perform cortical reconstruction and volumetric segmentation of the whole brain,^57,58^ the Freesurfer image analysis suite (version 7.1, http://surfer.nmr.mgh.harvard.edu) was used. The automated recon-all pipeline consists of more than 30 stages, including motion correction, intensity normalization, a Talairach transform of each subject’s native brain, removal of the non-brain tissue, segmentation of the subcortical grey matter/white matter (GM/WM) tissue and GM/WM boundary tessellation. The quality of the FreeSurfer segmentation was visually inspected by AT and RT, and scans with poor image quality or suboptimal segmentation were excluded, but images with subtle motion artefacts were retained.

Typically, each participant’s brain is registered to a standard template like fsaverage based on the gyral and sulcal patterns. This standardization enables comparison of cortical metrics across participants in a common space.^59^ Instead of using the fsaverage template, which is created based on adult brains, we created a custom group-average template to better represent the anatomical features of children and adolescents, including patients with CHD. The instructions for initializing the custom template with the FreeSurfer template can be found on this website (https://surfer.nmr.mgh.harvard.edu/fswiki/SurfaceRegAndTemplates). Each participant’s cortical surfaces were then registered to this custom group-average template, ensuring a more accurate alignment of cortical features across participants.

After registration to the template, CT, SA and GI values were extracted for each participant. CT was measured as the shortest distance between the pial surface and the GM/WM boundary, and the mean CT for each participant was computed by averaging CT values at each vertex for both hemispheres. SA was calculated as the total area of the cortical mantle on the pial surface. Local GI measures the proportion of cortical SA contained within sulcal folds compared with the external smoothed cortical surface.^29^ The entire cerebral cortex was then parcellated into 34 distinct regions per hemisphere using the Desikan–Killiany atlas,^60^ to create maps of volume, CT, SA and GI. Cortical maps for volume, SA and CT were smoothed using a 10 mm full-width at half-maximum (FWHM) surface-based Gaussian kernel to lower local anatomical variation and facilitate group-level statistical analysis. The GI map was smoothed using a 5 mm FWHM kernel, as local GI maps are already relatively smooth.^29^

### Statistical analysis

#### General statistical analysis

Statistical analyses of the whole-brain mean CT, total SA, mean GI and cortical volume were carried out using R software,^61^ version 4.3.2. Descriptive statistics comprised mean (standard deviation: SD) and median (interquartile range: IQR) for continuous variables and counts and proportions for categorical ones. Groups (such as the CHD and control groups) were compared using a two-sample *t*-test for continuous variables (e.g. age), a Mann–Whitney test for ordinal variables (e.g. parental education) or a *χ*^2^ test for categorical ones (e.g. sex). Missing parental education data (12 out of 100 in the CHD group and 12 out of 104 in the control group) were handled using group-wise median imputation. To assess potential bias, the subset of individuals without imaging data or those excluded due to artefacts was compared with the final sample demographics.

#### Statistical analysis for total/mean cortical structures

All the statistical analyses of the whole-brain measurements (volume, total SA, mean CT and mean GI) were controlled for age, sex and parental education. First, the interrelationships between cortical metrics were investigated using a Pearson’s correlation matrix. Then, multiple linear regression was used to test how total cortical volumes are associated with total SA, mean CT or mean GI (cortical volume ∼ [total SA or mean CT or mean GI] + group + age + sex + parental education) across the whole sample. To compare the predictive strength of the total SA, mean CT and mean GI in explaining cortical volume, we employed a bootstrapping approach. This analysis estimated the sampling distribution of standardized slopes (beta coefficients) 1000 times and their 95% confidence intervals (CIs) using the bias-corrected and accelerated method. By examining the overlap and width of these CIs, we determined whether the strength of association between cortical volume and SA differs significantly from that between cortical volume and CT or cortical volume and mean GI.

CHD-control group differences and group differences among further CHD subgroups (univentricular or biventricular; cyanotic or acyanotic) in cortical metrics (volume, SA, CT, GI) were evaluated using multiple linear regression. Interaction effects (group:age, group:sex and group:parental education) on cortical metrics were analysed through a multiple linear regression. Additionally, in the CHD group, an association between cortical metrics and the length of ICU stay was investigated, as this clinical risk factor has previously been identified as a predictive marker for neurodevelopmental outcomes.^62^

Furthermore, associations between cognitive outcomes (IQ and EF summary score; dependent variables) and cortical metrics (independent variables) were calculated using multiple linear regression. Interaction effects (cortical metrics:group) were tested to investigate if the strength of association between cortical metrics and cognitive outcomes differs between patients and controls. All the *P*-values for linear regressions were adjusted for multiple comparisons using the false discovery rate (FDR). Statistical significance was demarcated at a two-tailed *P* < 0.05.

#### MRI-based statistical analysis (GLM)

Group differences (CHD and controls) in regional-level cortical volumes, SA, CT and local GI were investigated using vertex-based multivariate General Linear Model (GLM) analysis, controlling for age and sex. A FreeSurfer-programmed ‘Different Onset, Different Slope’ (DODS) GLM was applied to the spatially normalized cortical data to create statistical maps depicting the group differences in cortical metrics, regressing out the effects of age and sex. Additionally, we covaried for total brain volume (TBV), total SA and mean CT separately to see if there are any disproportionately smaller or thinner regions, irrespective of cortex size/thickness in the CHD group.

To examine the cortical regions associated with IQ and EF across the entire sample, the same DODS model was used, incorporating additional IQ/EF covariates while adjusting for age, sex and group. Thereafter, interaction effects were investigated to determine whether group differences existed in the strength and direction of the association between cortical metrics and IQ/EF.

Monte Carlo simulation data, necessary for correcting multiple comparisons, was precomputed using 10 000 iterations to ensure an accurate estimation of the null distribution of cluster sizes. Then, cortical surface results were corrected for multiple comparisons using Freesurfer’s clusterwise correction, with a vertex-wise cluster-forming threshold of *P* < 0.05 (two-tailed), and adjusted for two hemispheres using the Bonferroni correction. The results with a 10 mm FWHM smoothing value (SA, CT and volume) and a 5 mm FWHM smoothing value (local GI) were reported, based on a previous report regarding clusterwise false positive rates when applying different FWHM values after the permutation simulation correction.^63^ As an additional validation approach, we re-ran all GLM analyses in a subsample with the best image quality only (without any minor movement artefacts, *n* = 111).

## Results

### Demographic data

Of the 204 participants who participated in the study (100 patients, 104 controls), 155 underwent cerebral MRI (60 patients, 95 controls). The main reasons for not undergoing cerebral MRI were implants in patients with CHD (e.g. pacemaker, stents, clips), or anxiety. Among those with MRI data, 26 (11 patients, 15 controls) were excluded due to poor image quality or suboptimal segmentation. Macroscopic brain abnormalities were reviewed; minor findings were detected in 5 patients, and no incidental findings were detected in controls (see Supplementary Text 1). As these incidental findings did not affect cortical segmentation, these subjects were not excluded from further analyses to maintain a representative sample.

Thus, the final sample size is *n* = 129 (49 patients, 80 controls) with a mean age of 13.2 years (SD = 1.3). The CHD group was older than the control group (*t*(df) = −3.56(117), *P* = 0.001). There was no difference in sex distribution between groups (*Χ*^2^(df) = 2.03(1), *P* = 0.154). The CHD group had lower parental education (*W* = 1006.5, *P* < 0.001), lower IQ (*t*(df) = −5.63(86), *P* < 0.001) and lower EF (*t*(df)=−4.83(95), *P* < 0.001) than controls. The sample characteristics of patients and controls are presented in Table 1, and the CHD diagnoses are listed in Supplementary Table 2.

**Table 1:** Demographic data of participants

Sample characteristics	CHD (*n* = 49)	Controls (*n* = 80)	*P*-value
Age, mean (SD)	13.65 (1.1)	12.86 (1.4)	0.001
Male sex, *n* (%)	31 (63)	39 (49)	0.154^a^
Parental education, median (IQR)	7 (6–9)	10 (8–12)	<0.001^b^
Gestational age, mean (SD), wk	39.40 (1.6)	39.06 (1.5)	0.310
Birth weight, mean (SD), g	3295 (559.9)	3174 (539.3)	0.580
IQ, mean (SD)	99.69 (11.6)	110.96 (9.7)	<0.001
EF summary score, mean (SD), *z*	−0.99 (1.1)	−0.06 (1.0)	<0.001
**CHD characteristics**			
Prenatal diagnosis, *n* (%)	9 (18)		
Univentricular CHD, *n* (%)	9 (18)		
Cyanotic CHD, *n* (%)	34 (69)		
**Perioperative time of first CPB surgery**			
Preoperative O_2_ saturation, mean (SD), %	87.17 (10)		
Age at surgery, mean (SD), month	2.58 (2.9)		
Lowest intrapoerative temperature, mean (SD), °C	28.94 (3.9)		
ECC time, mean (SD), min	170.27 (72.1)		
Length of hospital stay, median (IQR), days	21.00 (17–36)		
Length of ICU stay, median (IQR), days	7.00 (5–10)		
No. CPB surgeries, median (IQR)	1 (1–3)		
**Critical neurological or cardiac events ever**			
ECMO, *n* (%)	1 (2)		
Clinically relevant seizure, *n* (%)	2 (4)		
Stroke identified on cerebral MRI, *n* (%)	4 (8)		
Number of cardiac medications^c^ at assessment, median (IQR)	0 (0–4)		

CHD, congenital heart disease; ECC, extracorporeal circulation; ECMO, extracorporeal membrane oxygenation; ICU, intensive care unit; CPB, cardiopulmonary bypass; SD, standard deviation; IQR, interquartile range.

^a^Two-sampled *χ*^2^ test.

^b^Two-sampled Mann–Whitney U-test.

Others: two-sampled independent *t*-test.

^c^Cardiac medications are mainly ACE inhibitor, beta blocker, diuretic, aspirin.

The final sample did not differ from excluded participants (*n* = 75: 51 patients, 24 controls) regarding age (*t*(df) = −1.56(154), *P* = 0.121), sex (*Χ*^2^(df) = 0.00(1), *P* = 1.00), parental education (*W* = 5594, *P* = 0.060), IQ (*t*(df) = 1.87(110), *P* = 0.064), the proportion of cyanotic CHD (*Χ*^2^(df) = 0.33(1), *P* = 0.567) or univentricle CHD (*Χ*^2^(df) = 1.12(1), *P* = 0.290) and the length of ICU stay for patients (*t*(df) = −1.96(51.7), *P* = 0.055).

### Total/mean cortical alterations in CHD

**Figure 1:**
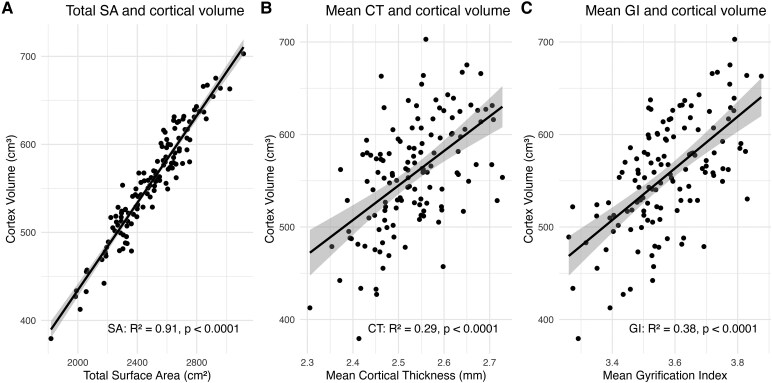
**(A–C) Comparison of the correlation strength of cortical volume and other morphometrics (total surface area, mean cortical thickness and mean gyrification index).** The scatterplots (**A–C**) show associations between cortical volume (cm^3^) and other morphological metrics across the whole sample (*n* = 129). Additional multiple regressions were conducted for each metric to estimate association strength, while adjusting for age, group, sex and parental education. (**A**) Cortical volume showed a statistically significant association with total SA (cm^2^) (*R*^2^ model fit of volume ∼ total SA = 0.914, *β* = 0.904, FDR-corrected *P* < 0.0001). (**B**) Cortical volume showed a statistically significant association with mean CT (mm) (*R*^2^ model fit of volume ∼ total SA = 0.298, *β* = 0.395, FDR-corrected *P* < 0.0001). (**C**) Cortical volume showed a statistically significant association with mean GI (*R*^2^ model fit of volume ∼ total SA = 0.383, *β* = 0.479, FDR-corrected *P* < 0.0001).

Correlation between total SA and mean GI was relatively high (*r* = 0.70), while correlations between total SA and mean CT (*r* = 0.29) and mean CT and mean GI (*r* = 0.22) were weak across hemispheres. Across the whole sample, total cortical volume correlated most strongly with total SA (*β* = 0.904), and showed moderate correlations with mean GI (*β* = 0.479) and mean CT (*β* = 0.395). Figure 1 shows the difference in correlation strength between cortical volume and total SA/mean CT/mean GI, with different *R*^2^ model fits (volume ∼ total SA: *R*^2^ = 0.914; volume ∼ mean CT: *R*^2^ = 0.289; volume ∼ mean GI: *R*^2^ = 0.383). Furthermore, bootstrapping revealed that SA has a stronger and more consistent relationship with cortical volume (95_CI: [0.938, 0.968]) compared to mean CT (95_CI: [0.398, 0.654]) or mean GI (95_CI: [0.513, 0.710]), as evidenced by the higher and narrower CI for SA. Moreover, the lack of overlap between the intervals of SA–CT and SA–GI suggests a potential difference in the predictive strength of these variables. For supplementary purposes, the cortical volume derived from segmentations and the cortical volume derived from surface reconstruction were compared, but there was no statistically significant difference between the two (*t*(df) = −1.72(256), *P* = 0.864), and they were highly correlated (Pearson’s correlation *r* = 0.999894, *P* < 2.2e−16).

**Figure 2:**
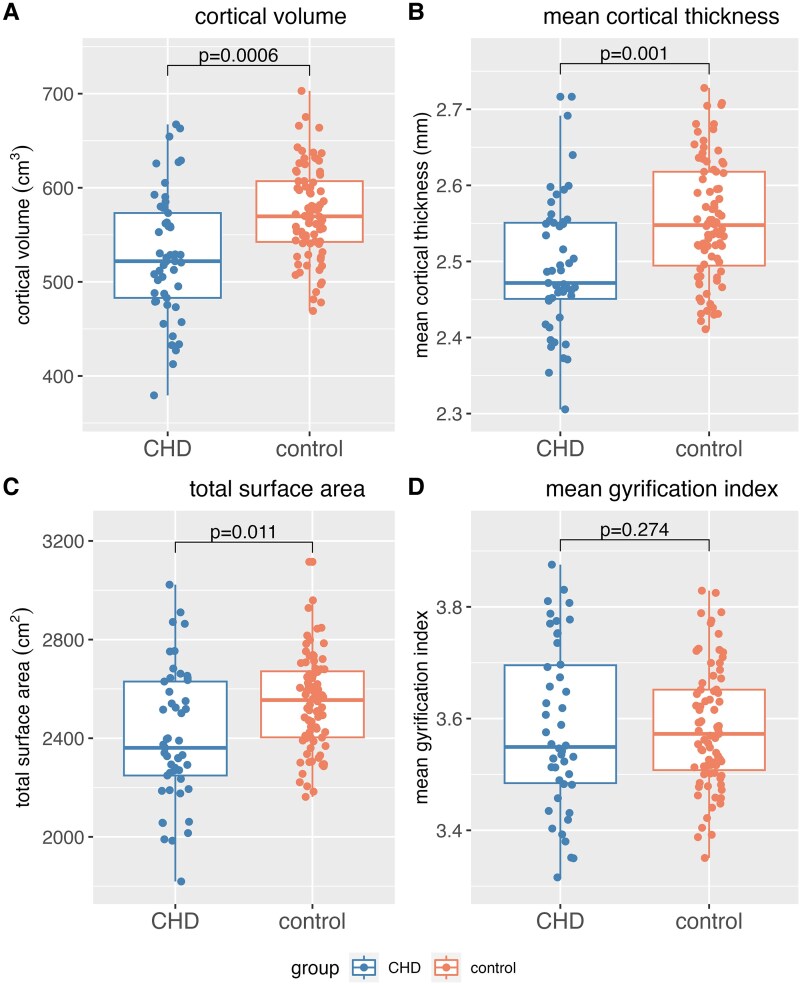
**(A–D) Comparison of the CHD and control group in cortical volume, mean cortical thickness, total surface area and mean gyrification index.** Blue = CHD (*n* = 49), Red = control (*n* = 80). The CHD group showed lower values than controls for (**A**) cortical volume (cm^3^) (*P* = 0.0006, *β* = 0.283), (**B**) mean cortical thickness (mm) (*P* = 0.001, *β* = 0.298) and (**C**) total surface area (cm^2^) (*P* = 0.011, *β* = 0.208) in a multiple regression analysis. (**D**) Gyrification index did not show a statistically significant difference between the groups (*P* = −0.095, *β* = 0.283). Covariates such as age, sex and parental education were accounted for in the model, and *P*-values were FDR-corrected.

Patients with CHD showed statistically significantly lower values in total/mean cortical metrics (i.e. cortical volume, total SA, mean CT) compared to controls after controlling for covariates and FDR correction (see Fig. 2). However, there was no statistically significant difference in mean GI between CHD and controls (*P* = 0.274). Statistical details are provided in Supplemental Table 3. Furthermore, there were no statistically significant interaction effects of group:age, group:sex and group:parental education on any cortical metrics, except for group: parental education effects on total SA (*β* = −0.773, *P* = 0.018), indicating the effect of parental education on total SA (but not volume, mean CT or mean GI) is weaker in controls than the CHD group.

Among the CHD group, patients with cyanotic CHD showed lower mean CT than patients with acyanotic CHD (*β* = 0.373, 95_CI: [0.104, 0.642], *P* = 0.025). Similarly, patients with univentricular defects showed lower mean CT than those with biventricular defects (*β* = 0.478, 95_CI: [0.248, 0.709], *P* = 0.001). However, no statistically significant difference was observed in cortical volume, total SA and mean GI between any of these subgroups. Furthermore, the length of ICU stay after the first CPB surgery was not associated with any cortical metrics (cortical volume: *β* = −0.087, mean CT: *β* = −0.082, total SA: *β* = −0.038, mean GI: *β* = 0.016).

### Regional cortical alterations in CHD

**Figure 3:**
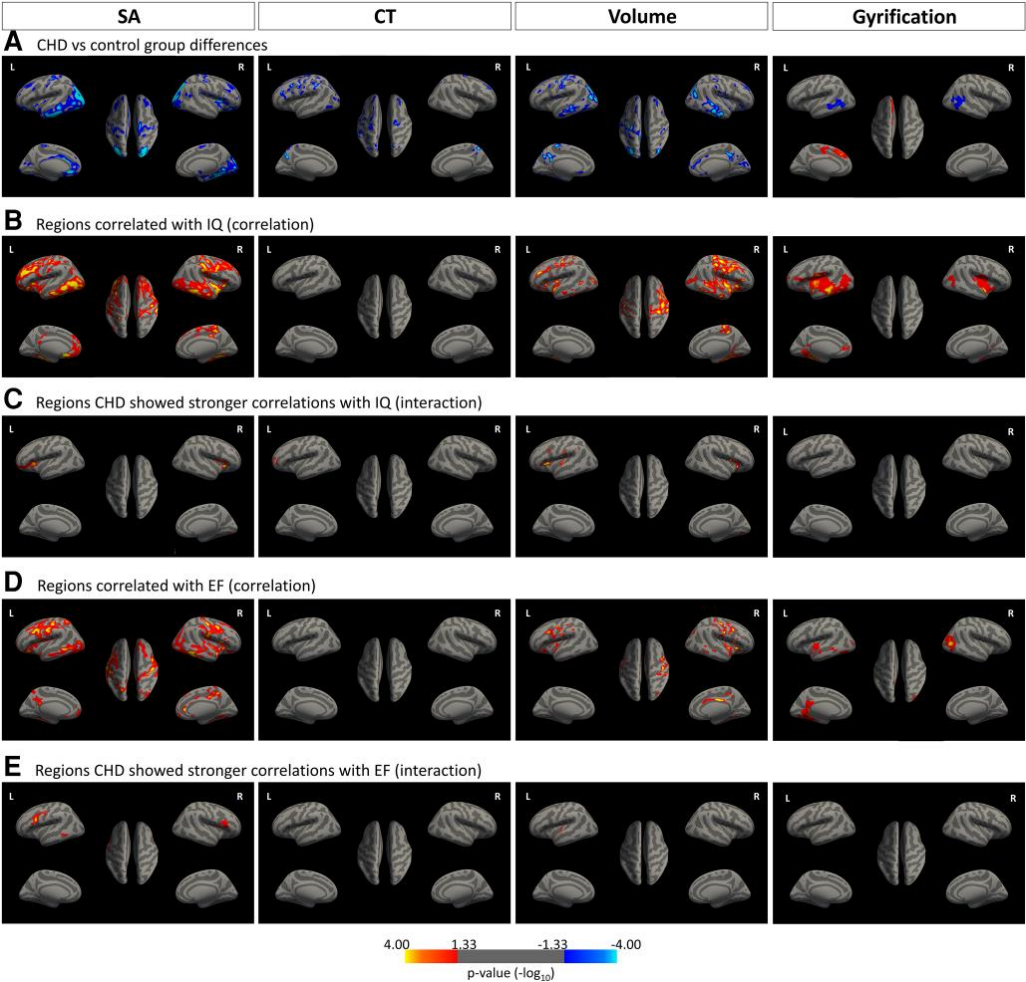
**(A–E) Cortical surface structures, comparison between groups and association with IQ/EF summary score.** All analyses were adjusted for age and sex, *P* < 0.05, clusterwise corrected and represented by blue clusters (negative correlation) or yellow-red clusters (positive correlation) across the whole sample (*n* = 129; CHD group = 49, control group = 80). The colour bar represents uncorrected significance values masked by the clusters that survived correction for multiple comparisons. SA, surface area; CT, cortical thickness; volume, cortical volume; GI, gyrification index; EF, executive function. (**A**) Patients with CHD showed significantly lower SA than controls in the left inferior parietal and postcentral, the right middle temporal and rostral medial frontal and supramarginal regions (the leftmost), significantly lower CT in the bilateral precuneus, precentral and rostral middle frontal, the left inferior temporal region (the second column from the left), and significantly lower volume in the left superior parietal, inferior temporal, precuneus, caudal middle frontal, lateral orbitofrontal, superior frontal and the right inferior parietal, lateral orbitofrontal, posterior cingulate and postcentral regions (the third column from the left). The CHD group showed higher local GI in left superior frontal regions, whereas in the bilateral middle temporal regions, CHD showed lower lGI than controls (the rightmost). (**B**) SA in the left medial orbitofrontal, isthmus cingulate and the right superior temporal regions were associated with IQ. No significant clusters associated with IQ were found for CT. For cortical volume, the bilateral fusiform, superior temporal and lateral occipital, the left rostral middle frontal and the right postcentral, lateral orbitofraontal and paracentral regions were associated with IQ. For GI, the left middle temporal, insula, the right superior temporal and inferior parietal regions were associated with IQ. No significant negative clusters (blue) were found. (**C**) The CHD group showed stronger associations between IQ and SA in the left pars triangularis, lingual and the right lateral orbitofrontal regions. For CT, the left rostral middle frontal region showed a stronger correlation with IQ in the CHD than in the control group. For volume, the left supramarginal, pars triangularis, caudal middle frontal and the right lateral orbitofrontal and lingual regions showed stronger associations with IQ. No statistically significant cluster was found for local GI. (**D**) SA in the left postcentral and the right pars opercularis regions, which were associated with the EF summary score. No significant clusters associated with the EF summary score were found for CT. For cortical volume, the bilateral postcentral, the left caudal middle frontal and fusiform, the right pars opercularis, posterior cingulate, lingual, inferior temporal and inferior parietal regions were associated with the EF summary score. For local GI, the left lingula and the right lateral occipital regions were associated with the EF summary score. No significant negative clusters (blue) were found. (**E**) The CHD group showed stronger associations between EF summary score and SA in the bilateral pars opercularis and the left lateral occipital regions. For CT and local GI, there were no clusters in which the group difference in the EF summary score was significant. For volume, the left superior temporal region showed stronger associations with the EF summary score than the controls.

After confirming a global reduction in cortical metrics (except for mean GI) in patients with CHD, we conducted a Freesurfer regional analysis to identify brain areas that are particularly affected in these patients. Comparisons of cortical SA, CT, GI and volume between patients with CHD and healthy controls (adjusted for age and sex) are shown in Fig. 3A. After clusterwise multiple testing corrections, the CHD group showed bilaterally significantly lower SA in 5 clusters (average cluster area size = 163.88 cm^2^), lower CT in 7 small clusters (average cluster area size = 25.26 cm^2^), lower cortical volume in 11 clusters (average cluster area size = 47.88 cm^2^) and lower local GI in 2 clusters (average cluster area size = 37.56 cm^2^), but also a higher local GI in one cluster (cluster area size = 59.35 cm^2^). The cluster statistics are presented in Table 2. For supplementary purposes to confirm imaging findings, the main results were compared with the selected subsample (*n* = 111) that had no minor movement artefacts; however, the results remained largely unchanged (See Supplemental Fig. 1).

**Table 2:** Cortical clusters comparison between the CHD and control group

Annotation	Side	Max	NVtx	Cluster size (cm^2^)	Clusterwise *P*-value	Talairach coordinates		
						*X*	*Y*	*Z*
**Surface area**								
Inferior parietal	L	−8.27	126 268	373.81	0.0002	−30	−74.6	18.8
Postcentral	L	−4.09	92 538	50.78	0.0002	−40	−28	49.3
Middle temporal	R	−6.00	105 454	238.57	0.0002	59.2	−21.6	−18.2
Rostral middle frontal	R	−4.50	121 751	134.62	0.0002	19.6	56	22.6
Supramarginal	R	−3.09	98 724	21.60	0.0056	49.5	−27.1	38.2
**Cortical thickness**								
Precuneus	L	−6.59	13 896	65.78	0.0002	−6	−71.6	30
Precentral	L	−4.01	129 230	46.26	0.0002	−56	−1.5	37.9
Rostral middle frontal	L	−4.09	48 842	20.34	0.0002	−43.6	33.4	24.8
Inferior temporal	L	−3.16	17 450	8.60	0.0365	−49.9	−64.5	−4
Precuneus	R	−6.31	71 304	17.55	0.0002	10.3	−60.5	53
Precentral	R	−3.82	137 882	9.43	0.0197	19.6	−17.2	62.5
Rostral middle frontal	R	−3.69	123 206	8.83	0.0316	30.2	35.3	31.9
**Cortical volume**								
Superior parietal	L	−6.67	157 742	125.72	0.0002	−28.4	−72	20.5
Inferior temporal	L	−5.89	7115	81.66	0.0002	−45.3	−30.5	−20.6
Precuneus	L	−5.39	136 087	22.80	0.0002	−13.5	−55.2	33.9
Caudal middle frontal	L	−3.41	123 129	16.51	0.0002	−34.5	4.9	35.9
Lateral orbitofrontal	L	−5.64	67 784	14.10	0.0004	−22.3	36.2	−11.8
Superior frontal	L	−3.80	65 524	12.60	0.0020	−13.9	52.2	31
Inferior parietal	R	−5.77	147 528	103.23	0.0002	35.6	−74.3	34
Lateral orbitofrontal	R	−3.72	50 935	71.62	0.0002	15.2	31.7	−23.3
Posterior cingulate	R	−4.10	141 645	38.44	0.0002	13.4	−38.3	38.3
Postcentral	R	−4.98	98 756	25.59	0.0002	38.1	−13.6	22.4
Postcentral	R	−2.90	140 326	14.35	0.0004	41	−33.8	55.7
**Gyrification index**								
Superior frontal	L	3.63	73 045	59.35	0.0002	−8.7	28.8	36.4
Middle temporal	L	−3.47	42 294	33.41	0.0044	−49	−65	−0.5
Middle temporal	R	−3.22	137 283	41.71	0.0002	59.2	−56.8	0.3

*P* < 0.05, corrected for multiple comparisons; Max, the maximum -log10(*P*-value) in the cluster; NVtx, the vertex number at the maximum.

As an additional analysis to examine the regional specificity of these findings, we covaried for the total SA to see if there are any disproportionately smaller regions irrespective of the cortex size for the CHD group. We found that the bilateral inferior temporal SA (left area size = 27.02 cm^2^, right = 20.19 cm^2^) and the right inferior parietal SA (area size = 25.75 cm^2^) were particularly smaller in patients with CHD compared to controls when total SA size was accounted for. For CT, there were no regions where group differences were significant when accounting for the mean CT. Interestingly, for local GI, the CHD group showed particularly higher local GI than the control group when controlling for TBV in the left inferior temporal, postcentral, the right paracentral and precentral regions. See Supplemental Fig. 2 for more details.

### Cognitive outcomes and cortical structures

On the global level, IQ was significantly associated with total SA, mean CT and cortical volume (mean CT showing the weakest association) across the whole sample. In contrast to IQ, the EF was associated only with cortical volume and total SA, not with mean CT. Mean GI was not statistically significantly associated with either IQ or EF. Statistical details are presented in Table 3. The interaction effect was non-significant, indicating that the strength of associations between IQ/EF and cortical metrics did not differ between groups.

**Table 3:** Multiple regression for IQ/EF summary score and cortical metrics across the whole sample (*n* = 129)

Effect	*B*	Standard error	95% CI	*β*	*P*-value	*R* ^2^	*P*-model fit
**IQ ∼ cortical volume**							
Cortical volume	0.084	0.018	0.257–0.590	0.423	1.01E−05		
Age	0.463	0.687	−0.096 to 0.199	0.051	0.686	0.351	1.39E−11
Sex	4.027	1.939	0.012–0.330	0.171	0.066		
Parental education	1.881	0.424	0.207–0.504	0.356	2.24E−05		
**IQ ∼ mean CT**							
Mean CT	30.733	11.585	0.063–0.388	0.225	0.0103		
Age	0.549	0.776	−0.106 to 0.228	0.061	0.669	0.274	1.02E−08
Sex	0.306	1.826	−0.137 to 0.163	0.013	0.867		
Parental education	2.633	0.415	0.363–0.632	0.498	6.37E−09		
**IQ ∼ total SA**							
Total SA	0.019	0.005	0.206–0.545	0.376	8.68E−05		
Age	0.123	0.688	−0.134 to 0.162	0.014	0.935	0.327	1.21E−10
Sex	3.735	1.994	−0.005 to 0.322	0.159	0.085		
Parental education	1.926	0.435	0.212–0.516	0.364	2.12E−05		
**IQ ∼ mean GI**							
Mean GI	10.365	8.045	−0.059 to 0.294	0.118	0.200		
Age	0.064	0.776	−0.160 to 0.174	0.007	0.935	0.242	1.24E−07
Sex	0.585	1.962	−0.137 to 0.186	0.025	0.867		
Parental education	2.531	0.432	0.337–0.620	0.478	5.60E−08		
**EF ∼ cortical volume**							
Cortical volume	0.006	0.002	0.154–0.527	0.340	0.001		
Age	0.035	0.070	−0.120 to 0.202	0.041	0.894	0.208	1.14E−06
Sex	0.319	0.203	−0.032 to 0.316	0.142	0.168		
Parental education	0.135	0.044	0.103–0.438	0.271	0.003		
**EF ∼ mean CT**							
Mean CT	1.532	1.186	−0.058 to 0.294	0.118	0.227		
Age	0.020	0.077	−0.155 to 0.203	0.024	0.874	0.145	1.01E**−**04
Sex	0.016	0.186	−0.154 to 0.168	0.007	0.931		
Parental education	0.195	0.042	0.240–0.542	0.391	1.36E−05		
**EF ∼ total SA**							
Total SA	0.002	0.0005	0.135–0.510	0.322	0.002		
Age	0.012	0.069	−0.144 to 0.174	0.015	0.874	0.201	1.90E**−**06
Sex	0.316	0.205	−0.036 to 0.317	0.141	0.169		
Parental education	0.136	0.045	0.103–0.441	0.272	0.003		
**EF ∼ mean GI**							
Mean GI	0.952	0.813	−0.073 to 0.299	0.113	0.244		
Age	0.012	0.076	−0.161 to 0.190	0.014	0.874	0.143	1.16E**−**04
Sex	0.063	0.196	−0.142 to 0.198	0.028	0.856		
Parental education	0.187	0.043	0.220–0.531	0.375	3.49E−05		

CT, cortical thickness (mm); SA, surface area (cm^2^); GI, gyrification index; CI, confidence interval; EF, executive function.

*P*-values are FDR corrected.

On the regional level, statistically significant positive associations were found between IQ and SA, local GI, as well as cortical volume, across the entire sample, but not with CT (see Fig. 3B). The association between IQ and SA was especially strong in the left medial orbitofrontal and right superior temporal regions. Furthermore, in several regions, the CHD group showed a stronger association with IQ across all cortical metrics, except local GI (see Fig. 3C). Interestingly, one CT cluster in the rostral middle frontal region remained significant in the group–IQ interaction analysis, indicating that the CHD group had stronger relationships between CT and IQ in this region, despite the absence of a CT–IQ association in the full sample. Cluster statistics are presented in Table 4.

**Table 4:** Cortical clusters statistics, association and interaction with IQ

Cortical clusters that are associated with IQ								
Annotation	Side	Max	NVtx	Clustersize (cm^2^)	Clusterwise *P*-value	Talairach coordinates		
						*X*	*Y*	*Z*
**Surface area**								
Medial orbitofrontal	L	5.77	154 316	472.12	0.0002	−8	19.9	−11.5
Isthmus cingulate	L	2.95	94 473	16.35	0.04723	−5.7	−45.8	22
Superior temporal	R	5.84	44 098	426.95	0.0002	47.1	−16.5	−2.8
**Cortical thickness**								
No significant clusters were found								
**Cortical volume**								
Rostral middle frontal	L	5.90	18 121	62.55	0.0002	−44.2	31.7	25.7
Lateral occipital	L	3.82	45 886	50.42	0.0002	−40.7	−80.9	9.4
Superior temporal	L	4.88	79 243	48.83	0.0002	−45.9	−6.2	−12.2
Fusiform	L	5.02	163 034	13.87	0.0006	−35.7	−42.2	−11.1
Rostral middle frontal	L	2.48	55 082	10.38	0.01017	−27.1	30.7	35.7
Postcentral	R	5.66	64 037	74.56	0.0002	32.2	−32.4	57.5
Superior temporal	R	4.99	63 175	50.10	0.0002	45.5	−16.9	−4.7
Fusiform	R	4.93	48 225	46.89	0.0002	36.4	−36.9	−17.4
Lateral orbitofrontal	R	5.79	1141	13.56	0.0012	29.2	24.2	0.9
Lateral occipital	R	2.61	129 166	9.65	0.01653	23.8	−100.8	1.4
Paracentral	R	4.92	150 120	8.65	0.03666	18.4	−42.8	45.1
**Gyrification index**								
Middle temporal	L	4.37	35 894	264.29	0.0002	−64.8	−34.4	−11.9
Insula	L	3.81	13 115	45.48	0.0004	−29.4	4.6	−12.6
Superior temporal	R	4.35	163 310	83.43	0.0002	55.1	6.4	−7.7
Inferior parietal	R	2.72	97 032	71.01	0.0002	44.9	−74.1	12.3

Cortical clusters that had a stronger association with IQ in CHD than controls								
Annotation	Side	Max	NVtx	Clustersize (cm^2^)	Clusterwise *P*-value	Talairach coordinates		
						*X*	*Y*	*Z*
**Surface area**								
Pars triangularis	L	3.88	16 896	54.10	0.0002	−29.2	22.4	13.7
Lingual	R	3.08	31 904	19.94	0.00679	16.6	−89.2	−9.1
Lateral orbitofrontal	R	4.89	27 185	19.36	0.00838	29.2	23.3	1.9
**Cortical thickness**								
Rostral middle frontal	L	3.57	141 255	13.19	0.0012	−38.8	42.3	4.2
**Cortical volume**								
Supramarginal	L	3.84	95 048	18.15	0.0002	−59	−21.3	24.3
Pars triangularis	L	5.50	38 015	14.89	0.0002	−28.2	25.8	10.6
Caudal middle frontal	L	3.13	66 571	8.84	0.03155	−32.6	3.4	27.5
Lateral orbitofrontal	R	4.54	51 070	11.00	0.00579	29.1	23.8	0.5
Lingual	R	2.66	70 001	8.77	0.03292	15.8	−90.7	−8.1
**Gyrification index**								
No significant cluster was found								

Max, the maximum −log10(*P*-value) in the cluster; NVtx, the vertex number at the maximum.

*P* < 0.05, corrected for multiple comparisons.

The EF was significantly associated with regional SA, local GI and cortical volume, but not with regional CT across the whole sample (see Fig. 3D). The association between the EF and SA was especially strong in the bilateral postcentral region. Significant interaction effects revealed that several regions in SA and volumes showed stronger associations with EF in patients with CHD than in controls. There were no significant CT or local GI clusters in this interaction analysis (see Fig. 3E). Cluster statistics are described in Table 5.

**Table 5:** Cortical clusters statistics, association and interaction with EF summary score

Cortical clusters that are associated with EF summary score								
Annotation	Side	Max	NVtx	Clustersize (cm^2^)	Clusterwise *P*-value	Talairach coordinates		
						*X*	*Y*	*Z*
**Surface area**								
Postcentral	L	5.44	80 831	376.00	0.0002	−51.7	−21	53.9
Pars opercularis	R	5.49	103 679	405.07	0.0002	41	14.4	11.2
**Cortical thickness**								
No significant clusters were found								
**Cortical volume**								
Caudal middle frontal	L	4.63	66 574	40.69	0.0002	−31.7	4.8	28.2
Postcentral	L	3.18	58 528	29.80	0.0002	−45.9	−9.8	14.5
Fusiform	L	2.72	90 524	17.53	0.0002	−39.8	−77.2	−15.5
Postcentral	R	4.59	19 998	54.10	0.0002	61	−9.6	27.7
Pars opercularis	R	5.19	72 960	33.42	0.0002	39.9	14.5	12
Posterior cingulate	R	5.77	146 637	16.87	0.0002	4.5	−13.9	31.5
Lingual	R	3.71	3905	15.13	0.0004	28.1	−57.6	−4.3
Inferior parietal	R	3.23	115 812	13.53	0.0014	35.3	−75.4	26.8
Inferior temporal	R	3.72	114 694	13.26	0.0014	59	−49.1	−15.2
**Gyrification index**								
Lingual	L	3.70	17 606	195.84	0.0002	−16.4	−47.6	−8.3
Lateral occipital	R	4.14	87 721	45.08	0.0002	47.6	−76	8.5

Cortical clusters that had a stronger association with EF summary score in CHD than controls								
Annotation	side	Max	NVtx	Clustersize (cm^2^)	Clusterwise *P*-value	Talairach coordinates		
						*X*	*Y*	*Z*
**Surface area**								
Pars opercularis	L	4.20	107 997	25.17	0.0024	−40.1	5.2	21.4
Lateral occipital	L	2.56	59 230	23.33	0.0042	−33.2	−86.4	−15
Pars opercularis	R	3.47	133 950	21.10	0.00619	39.2	4.4	14.2
**Cortical thickness**								
No significant clusters were found								
**Cortical volume**								
Superior temporal	L	2.57	125 308	12.11	0.0032	−53.4	−12.2	3.4
**Gyrification index**								
No significant clusters were found								

Max, the maximum −log10(*P*-value) in the cluster; NVtx, the vertex number at the maximum.

*P* < 0.05, corrected for multiple comparisons.

## Discussion

In the present study, we examined the global and regional cortical structures of adolescents with CHD and healthy controls to determine how cortical metrics (CT, SA, GI) are associated with the widely reported reduction of brain cortical volume and cognitive impairments in CHD populations. We found total and regional reductions in both SA and CT in the CHD group, with SA being more strongly associated with cortical volume reduction. While no group difference in mean GI was observed, the CHD group showed regional GI alterations compared with controls. Furthermore, across the whole sample, larger total and regional SA was strongly associated with higher IQ and EF. Mean CT was weakly associated with IQ but not with EF, and no significant associations were identified between regional CT and IQ/EF. Mean GI was not associated with IQ or EF, but regionally, a couple of local GI clusters were associated with IQ/EF. In several regions, the associations between cortical structures (SA, CT, Vol) and IQ/EF were stronger in patients compared to controls.

### Altered cortical morphometry in CHD

This is the first study to comprehensively investigate cortical morphological alterations and their relation to volumetric changes in CHD beyond childhood. Our results suggest that volumetric alterations are most strongly associated with SA, rather than CT or GI, in a sample including CHD, consistent with previous results in healthy adults and in CHD neonates.^35,42^ These findings provide more precise insights into how brain volume is affected in patients with CHD, given the distinct relationship between volume and each morphological component.

Furthermore, we demonstrated that adolescents with CHD showed significantly smaller total SA, and regionally in the left inferior parietal and postcentral regions, right middle temporal and rostral medial frontal and supramarginal regions, compared to controls. These detailed maps showing regional SA reductions are new, as previous morphometric studies in foetuses,^5^ neonates^16,33^ and adolescents^38^ all reported a reduction of total SA without regional specification and one adolescent study with lobe-based reduction of SA in temporal and parietal lobes, less so in frontal lobes, but not in occipital lobes.^20^

For the CT, we identified decreased mean CT in patients with CHD compared to controls, globally and regionally, in the bilateral precuneus, precentral and rostral middle frontal, and the left inferior temporal region. Findings from previous studies on CT in populations with CHD are inconsistent. One study in neonates^16^ and another in adolescents^20^ found no statistically significant group difference in mean CT, while another adolescent/adult study found significantly decreased CT.^38^ Another study by Cordina examined regional CT in 10 adult patients and reported a bilateral decrease in the dorsolateral prefrontal cortex, the precentral gyrus, the superior, inferior and supramarginal gyri, precuneus and the middle temporal gyrus.^40^ However, the regional CT reduction seen in our sample is less prominent than their results. This inconsistency in mean/regional CT results might stem from differences in sample size, age, CHD severity between the samples or from a lack of regional specificity, especially in mean CT studies.

For the mean GI, we did not find any statistically significant difference between CHD and controls, similar to one adolescent study,^38^ but inconsistent with most foetal/neonatal studies.^5,33-37^ For local GI, however, we identified lower local GI in the bilateral middle temporal region and higher local GI in the left superior frontal region. Cromb and colleagues suggested that lower GI was driven by a smaller TBV, as the difference between CHD and controls disappeared when TBV was accounted for.^34^ However, in our sample, after accounting for TBV, the CHD group showed higher local GI in multiple regions than controls. This result should be considered in the context of the intercorrelation between these neurodevelopmental parameters,^35^ and may reflect the relatively larger groupwise difference in TBV, which demonstrates a 7.89% reduction in adolescents with CHD (*P* < 0.001), relative to that of the mean GI, which is reduced by 0.43% in CHD (*P* = 0.55) in our data. The apparent differences in regional GI after TBV correction, which persist in adolescents in our sample but not in neonates,^34^ may also reflect an altered developmental trajectory during childhood, but longitudinal studies would be needed to clarify this.

Regarding comparison among CHD groups, we found that cyanotic CHD patients, compared to acyanotic CHD patients, and patients with univentricular defects, compared to those with biventricular defects, showed a statistically significantly lower mean CT, although the effect is relatively small. In contrast, no significant differences in SA, mean GI or cortical volume were identified between CHD subtypes. Furthermore, the length of ICU stay after the first CPB surgery, which is an indirect indicator of CHD severity, was not associated with any of the global cortical metrics. This was unexpected, as a number of studies suggest CHD severity tends to be negatively associated with brain volume.^3,64^ Our CHD sample was quite heterogeneous, so the cortical structural differences among patients with CHD should be confirmed with a larger sample.

### Mechanism of morphometric alterations in CHD

Different mechanisms may exist for reduced SA and CT seen in populations with CHD, as SA and CT are regulated by distinct genetic mechanisms,^24,25^ underscoring their independent developmental origins. SA is influenced by the number and spacing of cortical columns, determined by the proliferation of progenitor cells (symmetric division) in the subventricular zone (SVZ).^65-67^ In contrast, CT depends on the number of neurons within cortical columns, influenced by neurogenic (asymmetric) division.^65,66^ In addition to the number of neurons, CT is also influenced by the type and size of neurons, the dendritic and synaptic complexity, vascularization and the amount of neuropil and non-neuronal elements such as microglia and astrocytes.^68-70^ Consequently, alterations in distinct early developmental processes may have different effects on SA and CT, as evidenced in patients with CHD, who demonstrate a stronger reduction in SA than in CT. CHD-related risk may specifically affect the progenitor cells in the SVZ, which is crucial for later expansion of the SA.

Indeed, animal studies support this hypothesis; researchers have been investigating the precise effects of CHD-related hypoperfusion and hypoxia on cortical development at the cellular level. Morton induced chronic hypoxia in perinatal piglets and found that hypoxia reduces proliferation and neurogenesis in the SVZ. This depleted the pool of interneurons destined to populate the frontal cortex, limiting cortical expansion. This depletion of neuroblasts within the SVZ was replicated with post-mortem neonates born with CHD.^71^ Another study investigating neonatal piglets also showed that CPB surgery-induced insult alone impaired postnatal neurogenesis and migration to the frontal lobe, leading to an imbalance of excitatory/inhibitory interneurons and restricted cortical expansion/maturation.^72^ Given that SA is mainly influenced by the proliferation of neural precursor cells and formation of cortical columns, and CT is influenced not only by neurogenesis, we can assume the impact of CHD might appear more consistent and evident for SA than CT due to this difference in ontological trajectories. However, among patients with CHD, more severe types (cyanotic, univentricular) showed lower mean CT, but not in total SA or cortical volume, compared to milder types. Further investigations are needed as to why SA is more largely affected than CT in CHD as a whole, and why CT is affected especially among those with severe CHD types.

### Cortical structures and cognitive functions

#### Cortical structures and cognitive functions in healthy populations

Across the whole sample, larger total SA was associated with higher IQ/EF, with regional associations linking SA to IQ in medial orbitofrontal, cingulate and temporal regions and to EF in postcentral and inferior frontal areas. These findings align with a robust literature demonstrating positive SA–cognition associations across development. Prior studies have reported links between general cognitive ability and SA in prefrontal and temporal regions in children aged 4–12,^73^ genetically mediated associations between SA expansion and IQ across both hemispheres in youth,^74^ and region-specific associations between SA and crystallized (reading and vocabulary) and fluid intelligence (novel problem-solving) in children aged 9–11 and young adults.^43,44,75^ Despite heterogeneity in cognitive measures and implicated regions, the overall pattern of positive total and regional SA–cognition associations is highly consistent, supporting the robustness of our findings.

In contrast to SA, CT–cognition relationships were less consistent in our study. Mean CT was positively associated with IQ, but not with EF, and no significant regional CT–cognition associations were detected. This dissociation between mean and regional CT mirrors the mixed literature, in which many studies report no associations between mean or regional CT and intelligence across childhood, adolescence and young adulthood,^43,44,75,76^ while others report region-specific associations that vary in both direction and location. Positive CT–intelligence associations have been reported in frontal, temporal, parietal and occipital regions across wide age ranges,^73,77-79^ whereas other studies observed mixed positive and negative regional effects within the same sample^80^ or exclusively negative associations in cingulate, frontal and parietal regions in children and adults.^44,81^ Longitudinal work suggests these inconsistencies likely reflect strong age dependence: associations shift from negative to positive across childhood, reverse again in adulthood, or disappear in late adolescence.^46-48,78,82^ Our finding of a positive mean CT–IQ association without regional specificity in a cross-sectional sample aged 10–15 years may therefore have captured a limited aspect of the age-dependent evolving CT–cognition relationship.

While we did not find any significant associations between mean GI and cognitive functions, there were several region-specific clusters, where higher local GI was associated with IQ/EF. Our positive local GI–cognition findings align with the previous studies in healthy populations.^45,83,84^ However, the statistical significance level was much lower than with SA, and the areas in which local GI–IQ/EF relationships were observed mostly overlapped with those in which SA–IQ/EF associations were observed. Given the high correlation between SA and GI,^45,85^ our results are in line with a previous large-scale genetic study, which suggested that the GI–cognition relationship reflects, to a large extent, the SA–cognition relationship and the unique contribution of GI to cognitive ability is small.^86^

#### CHD-specific associations between cortical structures and cognitive functions

When investigated globally, higher total SA, mean CT and cortical volume were associated with better IQ in both patients and controls, without a significant group interaction. However, when examined regionally, cortical associations with IQ/EF were stronger in patients with CHD than in controls across several areas.

Our findings are comparable to a recent study by Abboud, which investigated the associations between cortical features and EF, as assessed using a self-rated short behavioural questionnaire, in CHD (aged 16–32).^38^ They identified that worse EF was associated with increased CT in the precuneus, posterior language areas, superior temporal, parietal, middle frontal and anterior cingulate cortex in patients with CHD. These areas overlap with networks critical for EF, like cognitive flexibility and decision-making. They also observed reduced SA in the lateral sulcus and increased local GI were associated with a worse EF score.^38^ It is intriguing that they found increased CT and local GI were linked with worse EF across the brain, while only one limited area of SA (lateral sulcus) was associated with EF. These results seem contradictory to our findings of strong SA/local GI associations with IQ/EF but not with CT. Another study identified a relationship between reduced CT and lower IQ in school-aged children with tetralogy of Fallot, in the left precuneus and right caudal middle frontal cortex.^41^ These previous findings and our results vary, possibly due to the dynamic change in CT–cognition relationship throughout the lifespan, as discussed previously. Given the consistent findings of positive SA–cognition relationships in previous studies of healthy populations, our sample with CHD is not an exception; thus, SA–cognition in CHD should be confirmed in future studies.

Furthermore, our findings of stronger associations between cortical structures and cognitive function in patients than in controls align with previous CHD studies, particularly regarding total/regional brain volumes.^20,87^ This suggests that although there are strong associations between cortical structures and cognitive function in healthy populations, the associations are even more apparent in populations with CHD, when brain growth is limited by a disease process.

#### Is SA a better indicator of cognitive function than CT?

Our results suggest that SA might be a better indicator for cognitive functions than CT, as correlations with IQ/EF were widely seen only in SA, but not in CT. Theoretically, higher cortical SA can enhance information processing capacity as larger SA equals a higher number of cortical columns, which are the functional units of the cortex.^22,88^ These additional columns allow for greater functional specificity of cortical columns and reduced overlap in neural representations, enabling the brain to process and store information more efficiently.^89^ This increased SA for distinct neural representations is also shown in the early visual cortex (V1 and V2), where visual SA correlated positively with neural population tuning sharpness and perceptual discrimination accuracy, while increased CT has the opposite effect.^90^ Furthermore, while CT contributes to cognitive function by reflecting interlaminar connections and synaptic remodelling, it might fluctuate depending on experience and learning,^91^ or show thinning during childhood due to the reflection of myelination.^92^ With previous literature on SA–cognition relationships and ontogeny, larger cortical SA seems to be the consistent underlying factor for better cognitive performance rather than higher CT.^44^

### Limitations

There are several limitations to this study. First, the CHD group was significantly older, and their parental education levels were lower than those of the control group, although we accounted for them in the statistical analysis. While our sample size was sufficient to detect CHD-control group differences in cortical metrics, the limited sample size and heterogeneity of CHD diagnoses affected the generalisability of this study. We created a custom template for our adolescents for better anatomical representation, more accurate registration and potentially more robust statistical analyses. However, the use of the custom template might have made our results study-specific and slightly less comparable to other studies using the standard fsaverage template. Furthermore, elucidating the longitudinal relationship between cortical structure and cognitive functions was beyond the scope of our cross-sectional study, although earlier studies in healthy populations indicated lifespan changes in the structure–function relationship.^46,82^ In future studies, our findings on the cortical structural association with cognitive outcomes in adolescents with CHD should be confirmed with a larger sample size, and changes in these associations over time should be assessed in longitudinal studies.

## Supplementary Material

fcag124_Supplementary_Data

## Data Availability

The de-identified data are available from the corresponding author upon reasonable request. The R Markdown file used for statistical analysis can be accessed from here: https://github.com/Asuka-Toyofuku/Cortical_analysis.git
