# Supplementary material for: Cortical morphology and cognitive impairments in adolescents with complex congenital heart disease
Source: Brain Commun. 2026 Apr 9;8(2):fcag124. doi: 10.1093/braincomms/fcag124 (PMC13095378; doi:10.1093/braincomms/fcag124)
Supplement: fcag124_Supplementary_Data [file fcag124_supplementary_data.docx]

**Supplemental Table 1**

**Neuropsychological Test Battery to Assess Executive Functions Performance**

| **Domains** | **Neuropsychological test** | **Test measurement** |
| --- | --- | --- |
| Working memory | Digit span forward and backward (WISC-IV) | No. of correct items |
|  | Letter-number sequencing (WISC-IV) | No. of correct items |
|  | Corsi block tapping test (Corsi) | No. of correct items |
| Inhibition | Inhibition Subtest interference, colour word interference task (D-KEFS) | Completion time |
|  | Go/NoGo (TAP) | No. of commission errors |
| Cognitive flexibility | Subtest letter-number-switching, trail making task(D-KEFS) | Completion time |
|  | TAP flexibility (TAP) | Median reaction time |
| Fluency | Subtests s-words and animals (RWT) | No. of correct items |
|  | Subtest filled-dots-only, design fluency test (D-KEFS) | No. of correct items |
| Planning | Tower task (D-KEFS) | Total achievement score |

**Supplemental Table 2**

**Diagnosis of congenital heart disease**

| Diagnosis | number |
| --- | --- |
| Aortic stenosis valvular | 1 |
| Atrioventricular septal defect (AVSD) | 1 |
| Complete AVSD | 2 |
| Coarctation of the aorta (CoA) | 2 |
| d-Transposition of the great arteries (d-TGA) | 16 |
| Double inlet left ventricle (DILV) | 1 |
| Ebstein's anomaly (severe) + Pulmonary stenosis (PS) | 1 |
| Hypoplastic left heart syndrome (HLHS) | 4 |
| Pulmonary atresia (PA) | 1 |
| PA with intact ventricular septum | 1 |
| PA with VSD and aortopulmonary collaterals (MAPCAs) | 1 |
| Pulmonary stenosis valvular | 1 |
| Tricuspid atresia (TA) | 1 |
| Truncus arteriosus type 1 | 2 |
| Total anomalous pulmonary venous connection (TAPVC) | 2 |
| Tetralogy of Fallot (TOF) | 4 |
| ventricular septal defect (VSD) doubly committed | 1 |
| Perimembranous VSD | 6 |
| Subaortic VSD | 1 |

**Supplemental Table 3**

**Group difference (CHD vs. controls) in cortical metrics**

| **Effect** | **B** | **Standard error** | **95% CI** | **β** | **p-value** |
| --- | --- | --- | --- | --- | --- |
| **Cortical volume** | |  |  |  |  |
| group | 34.833 | 9.483 | 0.136 ~ 0.430 | 0.283 | 5.67E-04 |
| age | -5.950 | 3.204 | -0.270 ~ 0.006 | -0.132 | 0.075 |
| sex | -56.627 | 8.223 | -0.597 ~ -0.347 | -0.472 | 4.06E-10 |
| Parental education | 7.000 | 1.993 | 0.119 ~ 0.406 | 0.262 | 8.29E-04 |
| **mean CT** |  |  |  |  |  |
| group | 0.053 | 0.016 | 0.133 ~ 0.464 | 0.298 | 0.001 |
| age | -0.023 | 0.005 | -0.500 ~ -0.200 | -0.350 | 4.43E-05 |
| sex | -0.027 | 0.014 | -0.305 ~ -0.004 | -0.155 | 0.050 |
| Parental education | -0.003 | 0.003 | -0.239 ~ 0.091 | -0.074 | 0.386 |
| **total SA** |  |  |  |  |  |
| group | 99.159 | 37.750 | 0.056 ~ 0.361 | 0.208 | 0.011 |
| age | -13.423 | 12.754 | -0.220 ~ 0.065 | -0.077 | 0.295 |
| sex | -224.039 | 32.735 | -0.610 ~ -0.357 | -0.483 | 4.20E-10 |
| Parental education | 31.404 | 7.935 | 0.159 ~ 0.451 | 0.305 | 2.03E-04 |
| **mean GI** |  |  |  |  |  |
| group | -0.026 | 0.024 | -0.263 ~ 0.073 | -0.095 | 0.274 |
| age | -0.037 | 0.008 | -0.517 ~ -0.222 | -0.369 | 1.64E-05 |
| sex | -0.079 | 0.021 | -0.443 ~ -0.153 | -0.298 | 2.11E-04 |
| Parental education | 0.012 | 0.005 | 0.049 ~ 0.372 | 0.210 | 0.016 |

Note: CT = cortical thickness (mm), SA = surface area (cm^2^), GI = gyrification index, CI = confidence interval, p-values are FDR corrected.

**Supplementary text 1: Incidental MRI findings in patients**

The following findings were reported for separate participants: 1) small anterior temporal encephalocele, 2) small post-ischemic/post-hemorrhagic defect communicating with the right lateral ventricle, 3) post-ischemic parenchymal defect involving the left caudate nucleus, 4) right frontal periventricular nodular heterotopia, 5) possible parietal periventricular gliosis

**
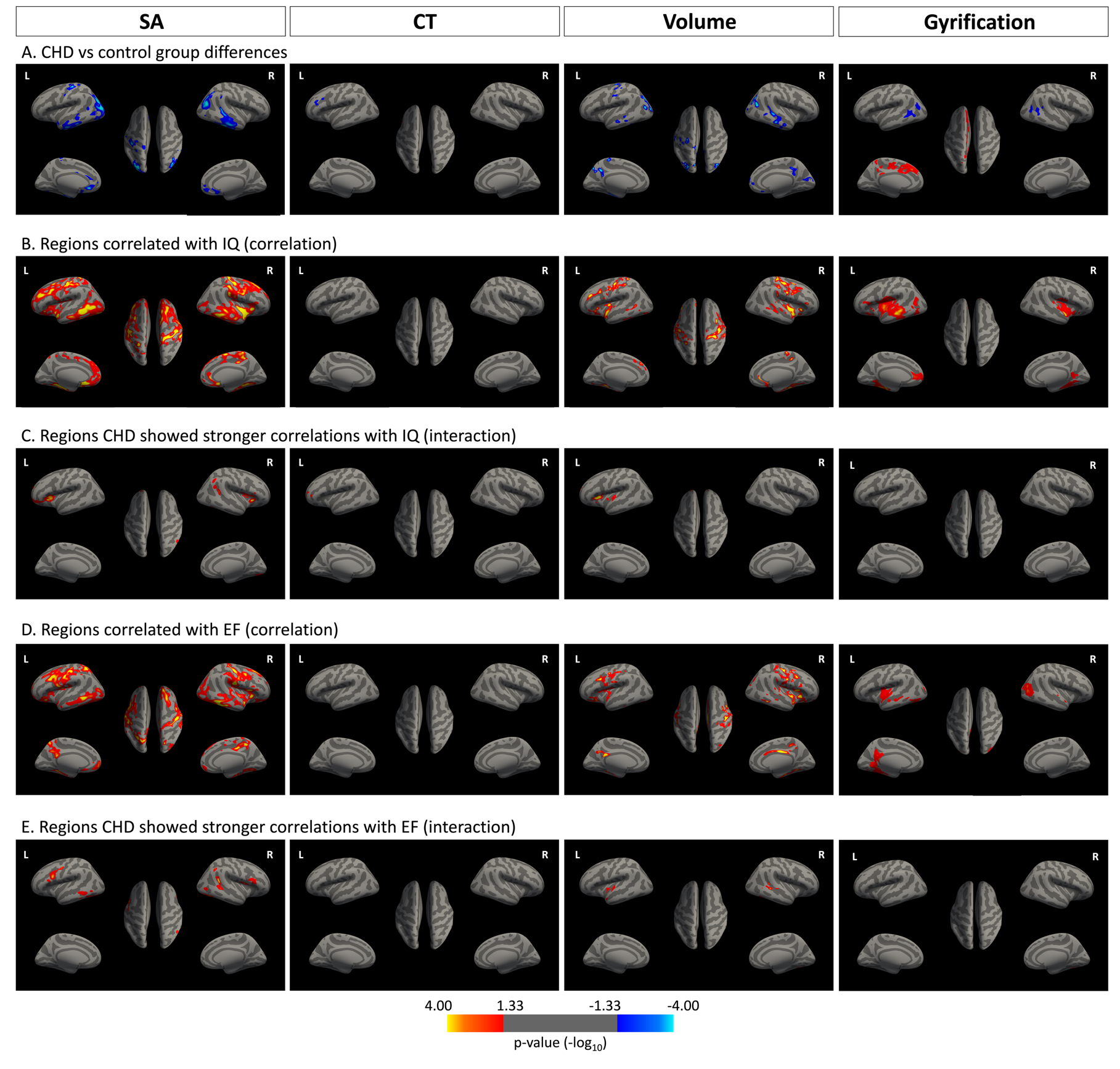
**

**Supplemental Figure 1**

**Cortical surface structures with restricted subsample without any minor movement artefacts (N=111)**

This selected sample did not differ significantly compared to the main result (N=129), but the group differences turned out less widespread, especially for the CT. All analyses were adjusted for age and sex, p < 0.05, cluster-wise corrected, represented by blue clusters (negative correlation) or yellow-red clusters (positive correlation). The colour bar represents uncorrected significance values masked by the clusters that survived correction for multiple comparisons. SA = surface area, CT = cortical thickness, Volume = cortical volume, GI = gyrification index. EF = executive function

**A.** Patients with CHD showed significantly lower SA than controls in the bilateral inferior temporal and medial orbitofrontal, left postcentral, the right inferior parietal regions (the leftmost), significantly lower CT in the left rostral middle frontal region (the second column from the left), and significantly lower volume in bilateral middle temporal and precuneus, the left superior parietal, precentral and inferior temporal, the right inferior parietal, cuneus, lateral orbitofrantal, superior frontal regions (the third column from the left). The CHD group showed higher local GI in left superior frontal regions, whereas in the left middle temporal and the right inferior parietal regions, CHD showed lower lGI than controls (the rightmost).

**B.** SA in the left medial orbitofrontal, the right superior temporal and rostal anterior cingulate regions were associated with IQ. No significant clusters associated with IQ were found for CT. For cortical volume, the bilateral fusiform, superior temporal, the left rostral middle frontal, lateral occipital, middle temporal, superior frontal region and the right superior temporal regions were associated with IQ. For local GI, the left fusiform, rostral anterior cingulate, the right superior temporal and precuneus regions were associated with IQ. No significant negative clusters (blue) were found.

**C.** The CHD group showed stronger associations between IQ and SA in the bilateral lateral orbitofrontal region and the lingual, and inferior parietal regions. For CT, the left frontal pole showed a stronger correlation with IQ in the CHD than in the control group. For volume, the left pars triangularis and rostral middle frontal regions showed stronger associations with IQ. For local GI, there were no clusters in which the group difference in the IQ was significant.

**D.**  SA in the bilateral postcentral region was associated with the EF summary score. No significant clusters associated with EF summary score were found for CT. For cortical volume, the left caudal middle frontal, lateral orbitofrontal, fusiform, isthmus cingulate, the right pars opercularis, parahippocampal, posterior cingulate, inferior temporal, inferior parietal regions were associated with the EF summary score. For local GI, the bilateral lingual and the right inferior parietal and superior temporal areas were associated with the EF summary score. No significant negative clusters (blue) were found.

**E.** The CHD group showed stronger associations between EF summary score and SA in the left middle temporal, pars opercularis, the right inferior parietal and precentral regions. For CT and local GI, there were no clusters in which the group difference in the EF summary score was significant. For volume, the left superior temporal and right bankssts and fusiform region showed stronger associations with EF summary score than controls.

**A.**


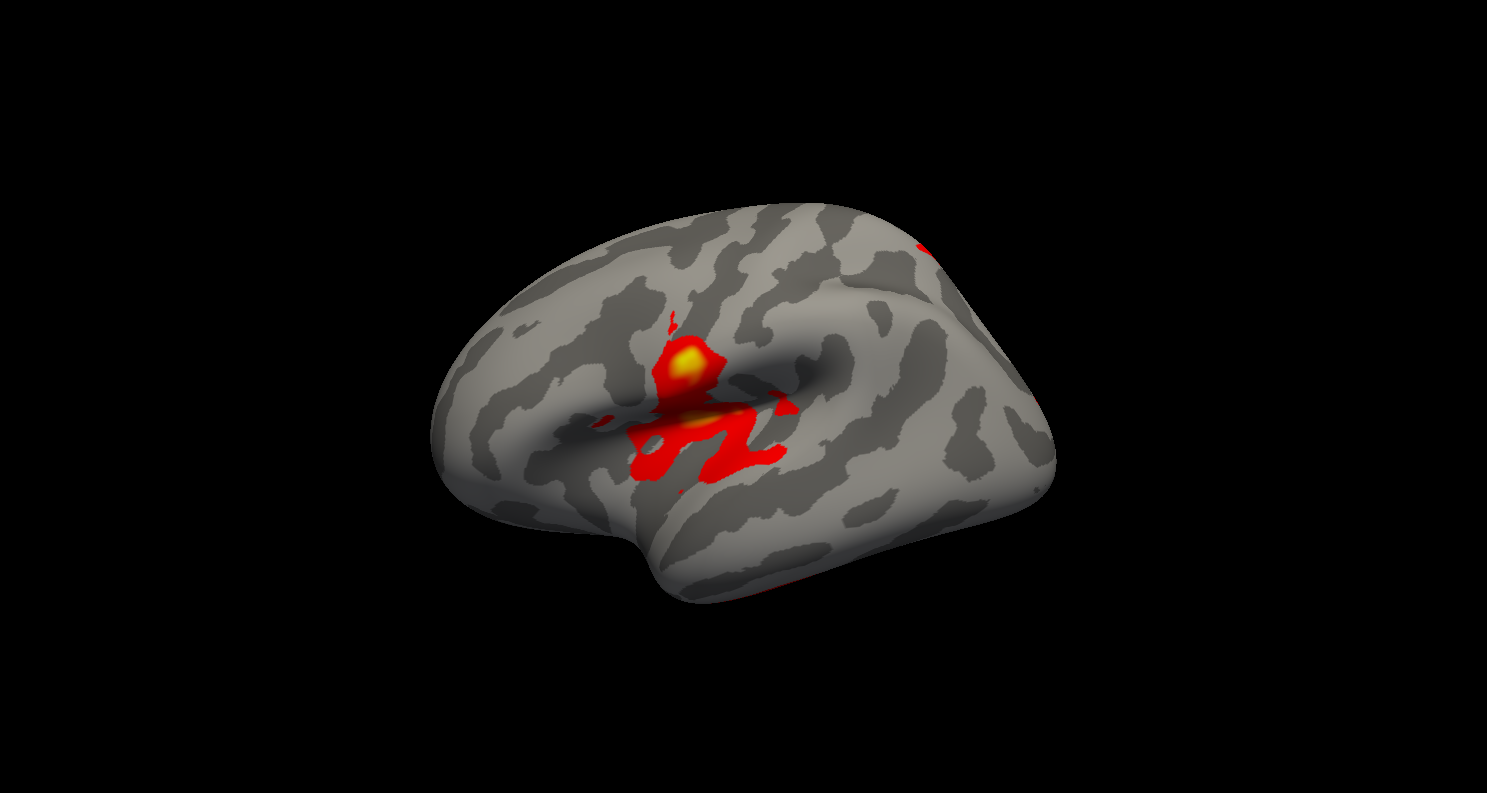

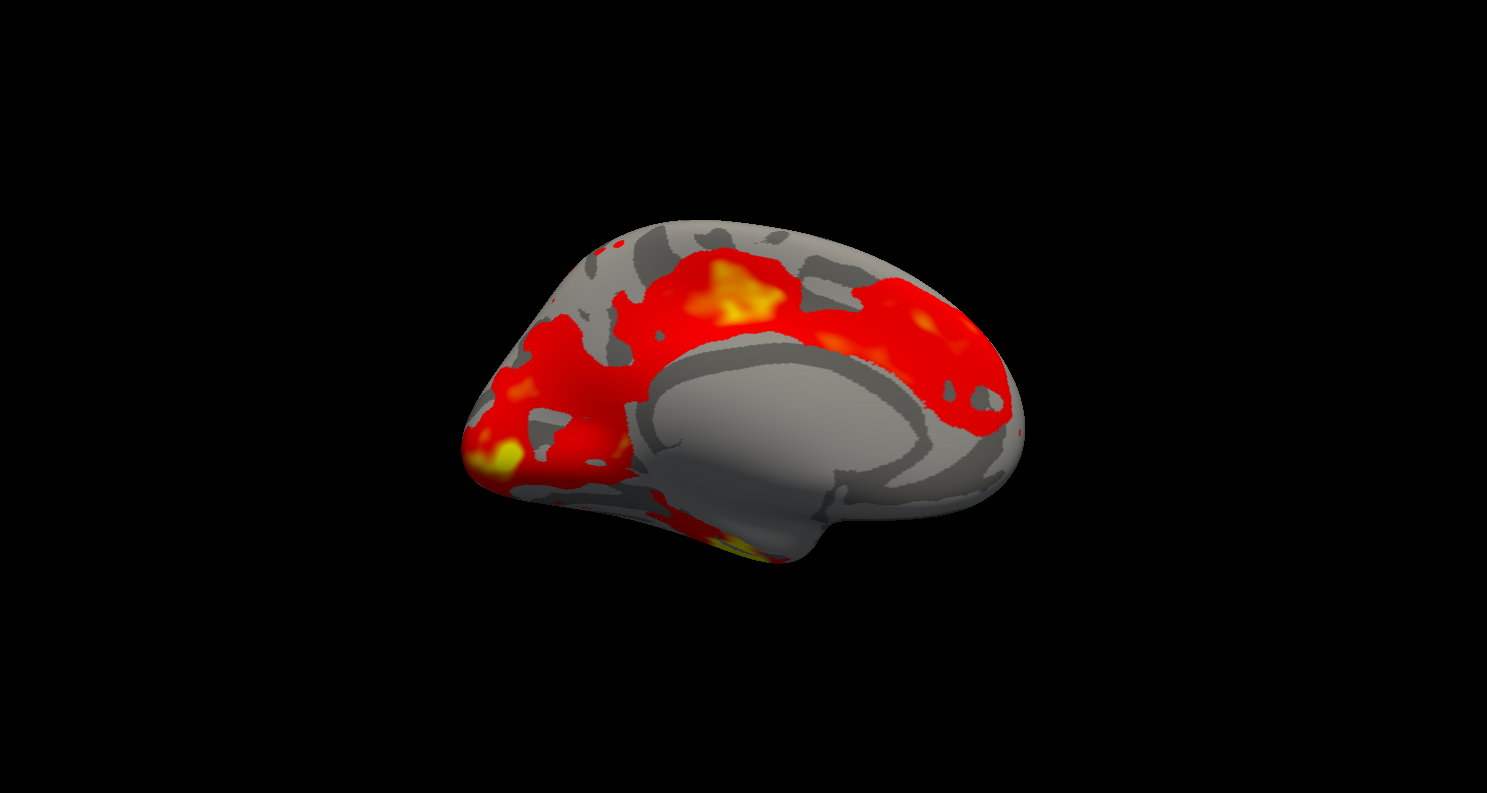

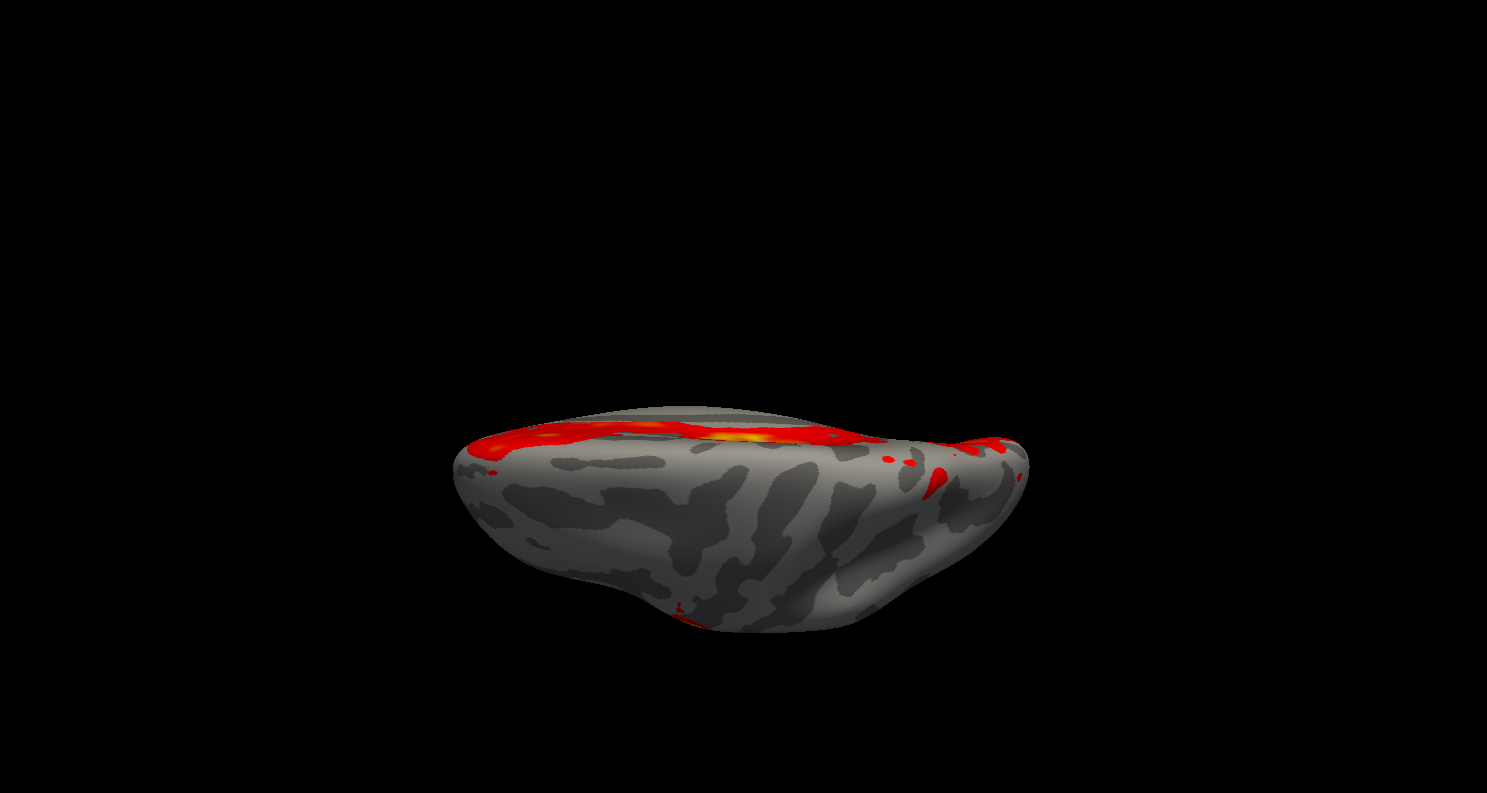

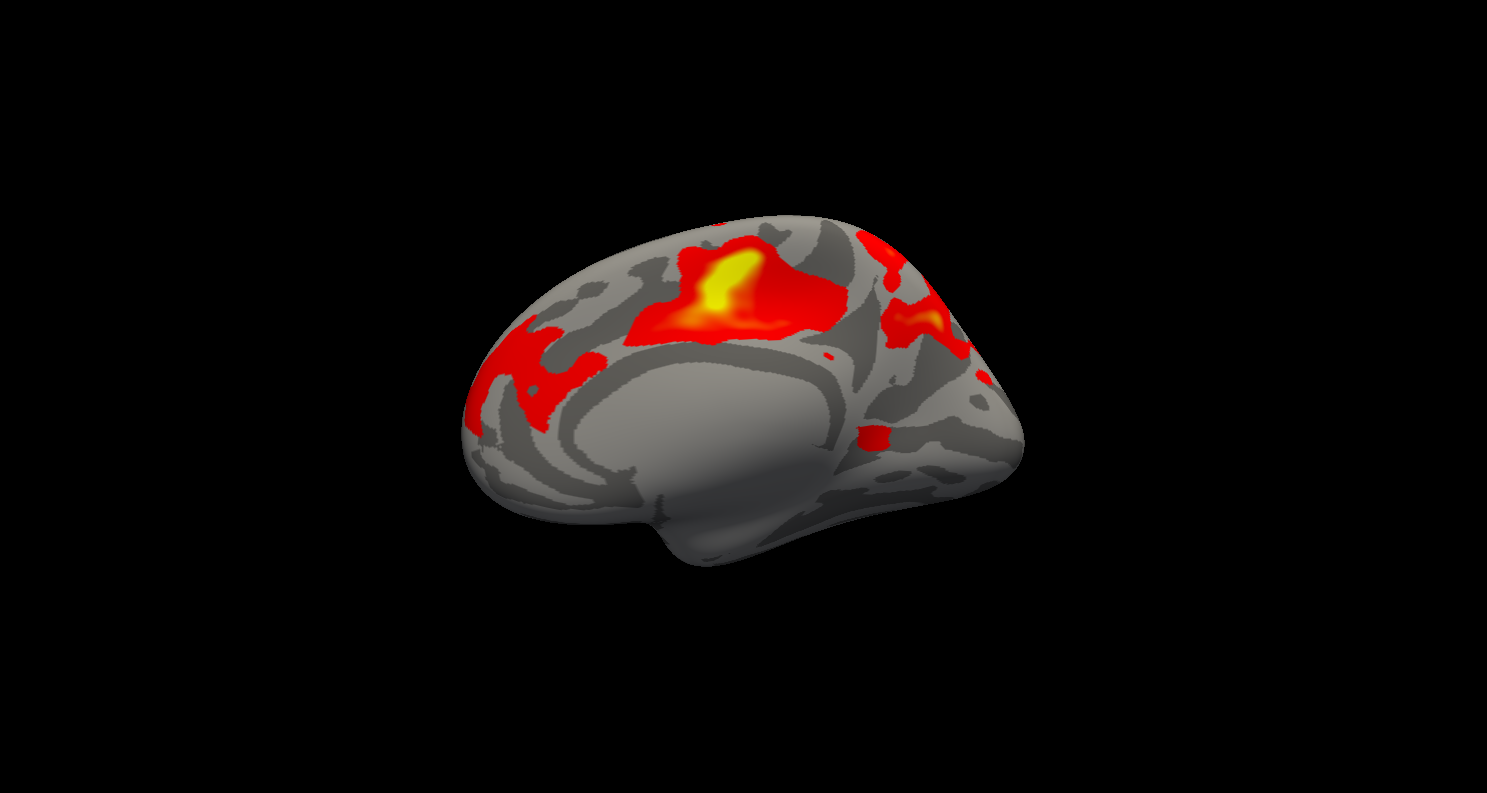

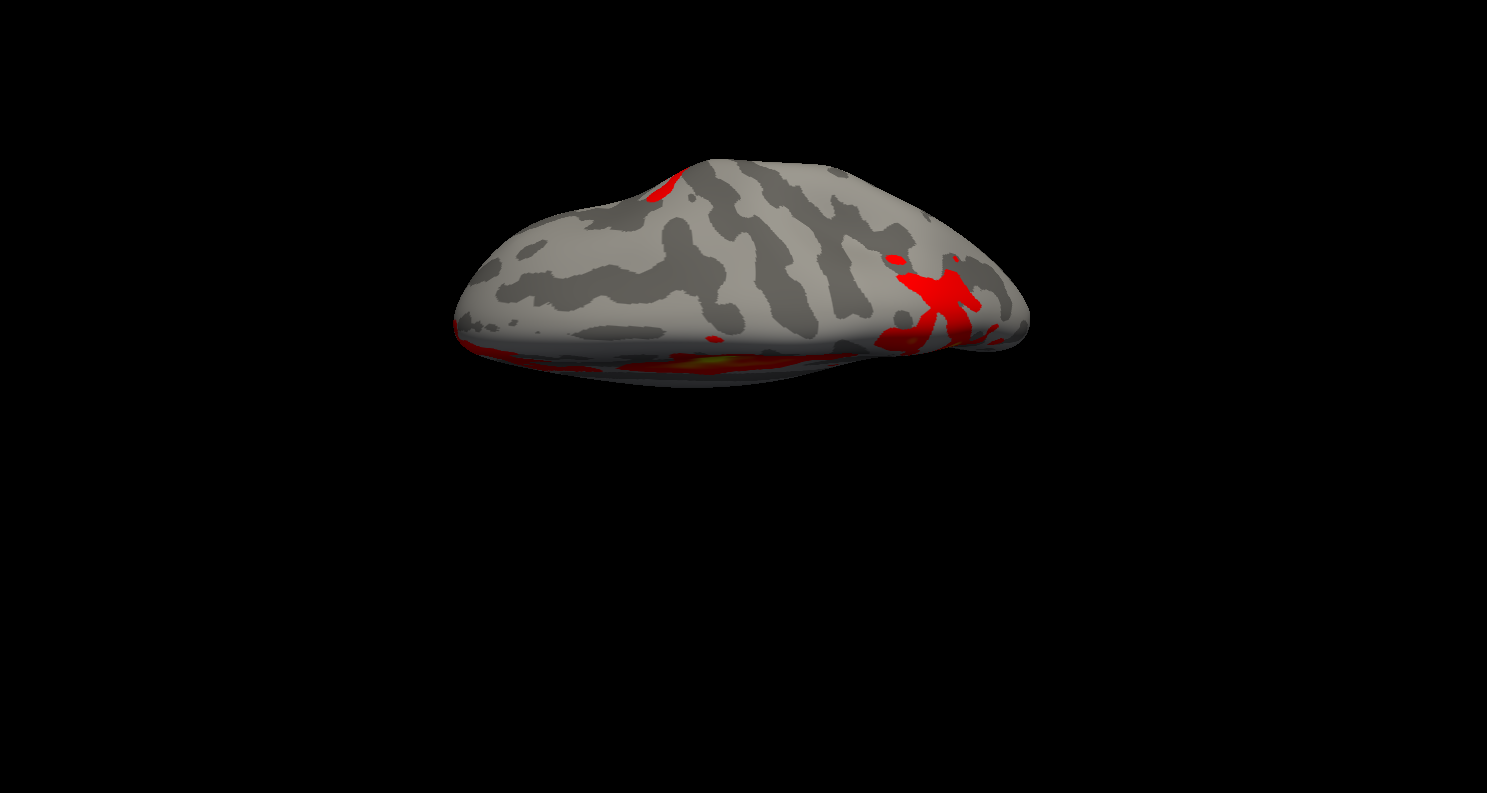

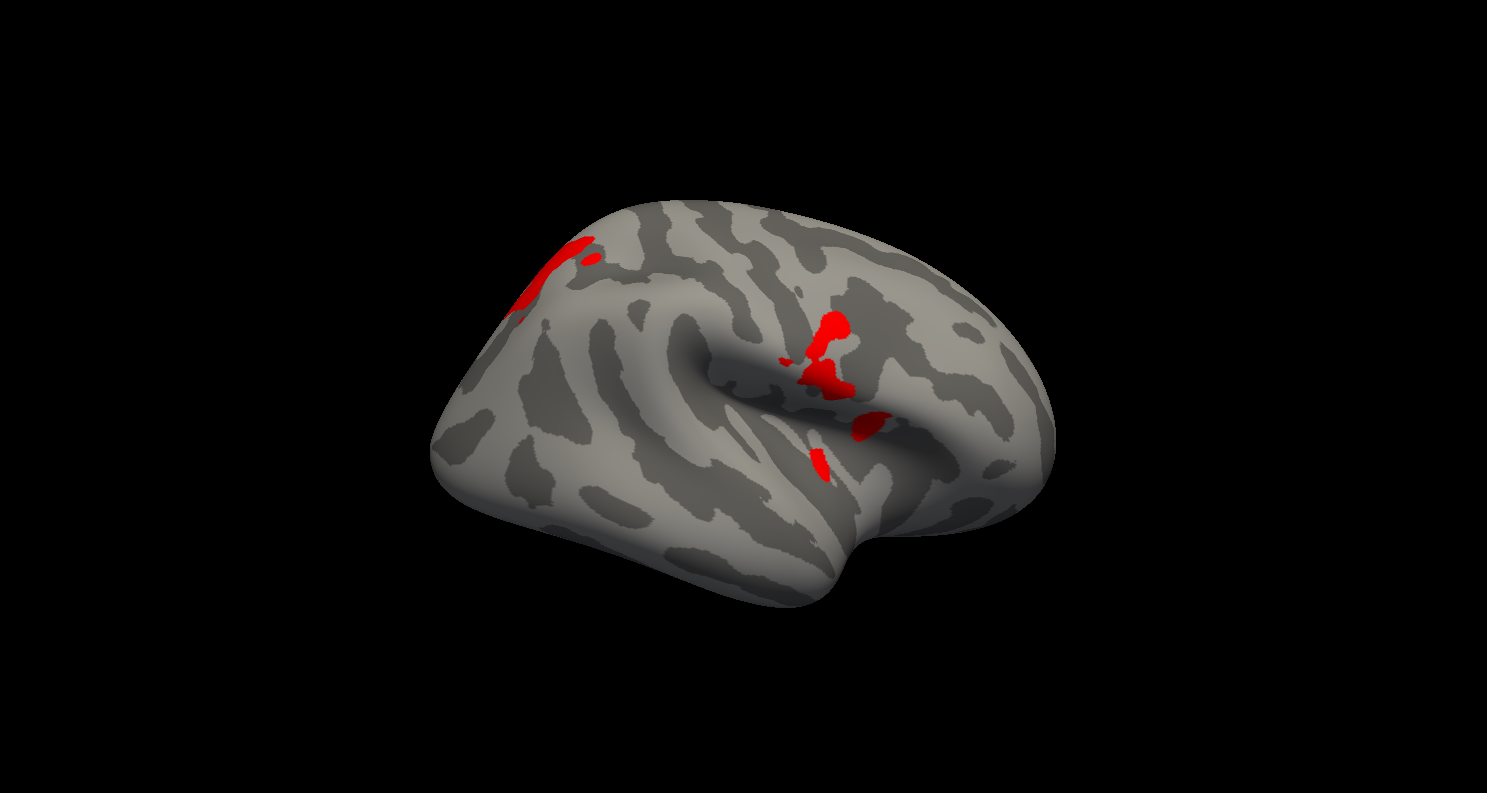


**R**

**L**

p-value (-log_100_)

-4.00

4.00

1.33

-1.33

**B.**

| **Group differences accounting for the total brain volume** | | | |  |  |  |  |  |
| --- | --- | --- | --- | --- | --- | --- | --- | --- |
|  |  |  |  |  |  | Talairach coordinates | | |
| Annotation | side | Max | NVtx | cluster size (cm^2^) | clusterwise p-value | X | Y | Z |
| **Gyrification index** |  |  |  |  |  |  |  |  |
| inferior temporal | L | 5.49 | 127763 | 239.02 | 0.0002 | -42.5 | -13.6 | -31.3 |
| postcentral | L | 3.97 | 133111 | 74.33 | 0.0002 | -56.4 | -7.2 | 19.4 |
| paracentral | R | 4.59 | 12200 | 164.72 | 0.0002 | 9.7 | -17.9 | 49.6 |
| precentral | R | 2.53 | 133842 | 43.88 | 0.0002 | 51.9 | 0.5 | 33.3 |

**Supplemental Figure 2**

**The CHD vs control group difference in local gyrification index when accounting for the total brain volume (N=129, final sample)**

A. Freesurfer cortical structure map for local GI. The analyses were adjusted for age, sex and total brain volume, p < 0.05, cluster-wise corrected, represented by blue clusters (negative correlation) or yellow-red clusters (positive correlation). The colour bar represents uncorrected significance values masked by the clusters that survived correction for multiple comparisons. The CHD group showed particularly higher local GI than the control group when controlling for total brain volume in the left inferior temporal, postcentral, the right paracentral and precentral regions (area sizes = 239.02cm^2^, 74.33 cm^2^, 164.72 cm^2^, 43.88 cm^2^, respectively).

B. Cluster statistics on the group differences in local GI when accounting for the total brain volume. p < 0.05, corrected for multiple comparisons; MAX = the maximum -log10(p-value) in the cluster, NVtx = the vertex number at the maximum.
